# Phenotype of p53 wild-type epitope-specific T cells in the circulation of patients with head and neck cancer

**DOI:** 10.1038/s41598-018-29067-5

**Published:** 2018-07-16

**Authors:** Andreas E. Albers, Xu Qian, Andreas M. Kaufmann, Daphne Mytilineos, Robert L. Ferris, Thomas K. Hoffmann, Albert B. DeLeo

**Affiliations:** 1 Department of Otorhinolaryngology, Head and Neck Surgery, Charité-Universitätsmedizin Berlin, corporate member of Freie Universität Berlin, Humboldt-Universität zu Berlin, and Berlin Institute of Health, Campus Benjamin Franklin, Berlin, Germany; 2 Clinic for Gynecology, Charité-Universitätsmedizin Berlin, corporate member of Freie Universität Berlin, Humboldt-Universität zu Berlin, and Berlin Institute of Health, Campus Benjamin Franklin, Berlin, Germany; 3 Department of Otolaryngology - Head and Neck Surgery, University of Pittsburgh Medical Center, Pittsburgh, PA USA; 4 Department of Otolaryngology, Head and Neck Surgery, University of Ulm, Ulm, Germany; 5 University of Pittsburgh Cancer Institute, Pittsburgh, PA USA; 6 Department of Pathology, University of Pittsburgh, Pittsburgh, PA USA

## Abstract

CD8^+^ cytotoxic T-cell (CTL) specific for non-mutated, wild type (wt) sequence p53 peptides derived from wt or mutant p53 molecules expressed in head and neck squamous cell carcinomas (HNSCC) have been detected in the circulation of patients with this disease. The frequency and differentiation/maturation phenotypes of these anti-tumor specific CTL can reflect the host’s immunologic response. Therefore, we investigated the frequency and phenotypes of wt sequence p53 peptide-specific CTL in patients with HNSCC (n = 33) by flow cytometric analysis using HLA-A*0201 tetrameric peptides (tet) complexed with the wt sequence p53_264–272_ or p53_149–157_ peptide and co-staining with phenotypic markers. One main finding was that increasing frequencies of tet^+^ CD8^+^ T cells in patients’ circulation correlated with increased frequencies of inactive naïve tet^+^ cells, while those with effector memory and terminally differentiated phenotypes, which are associated with positive anti-tumor immune responses, decreased. We also found that the frequency of circulating tet^+^ CD8^+^ T cells negatively correlated with p53 expression in tumor tissues and tumor stage. Our findings support further clinical-based investigations to define the frequencies and phenotypes of wt sequence p53 peptide-specific CD8^+^ T cells to predict disease severity, enhance selection of patients for inclusion in vaccination trials and highlight prerequisites to enhance immune susceptibility by activation of inactive naïve tet^+^ T cells and/or enhancing circulating effector T cell activity by checkpoint blockage.

## Introduction

The development and clinical application of novel biopharmaceutical agents targeting elements of the immune system, such as CTLA-4 and programmed death-1 (PD-1) checkpoint receptors as well as tumor associated cell surface antigens, has revolutionized immunotherapy and the oncologic treatment landscape. Patients with head and neck squamous cell carcinoma (HNSCC) are known to be immunosuppressed. Signaling defects in regulatory T cells (Treg) and cytolytic T lymphoctes (CTL) as well as a higher proportion of apoptotic T cells in these populations, in particular, anti-tumor specific CTL are detected in the peripheral blood of HNSCC patients compared to healthy individuals^1–3^. Thus, judiciously selected T-cell defined epitopes for cancer vaccines have been developed and defined with the aim to induce robust host anti-tumor immunogenicity. TP53, highly frequently mutated gene in HNSCC^4^, has been an attractive candidate for vaccines potentially capable of inducing immune responses in HNSCC patients directed against tumor-specific antigens. Mutant p53 protein, which accumulates in most HNSCC cells, potentially can yield mutation-specific p53 peptides. Although these epitopes would be tumor-specific, they have limited clinical applicability due primarily to the constraints imposed by antigen processing and presentation. In contrast, non-mutated, wild type (wt) sequence peptides derived from genetically altered p53 molecules in tumors have a greater potential of being processed and presented and represent a more practical approach for developing broadly applicable p53-based cancer vaccines for the prevention and treatment of HNSCC^5,6^.

Previously, we have demonstrated *in vitro* that the presentation of wt sequence p53 peptides pulsed on autologous-derived dendritic cells (DC) induced peptide-specific immune responses from peripheral blood lymphocytes obtained from HLA-A2^+^ normal donors as well as patients with HNSCC^7–10^. Dendritic cells (DC)-based wt sequence p53 peptide vaccines have been used for immunotherapy in a variety of human cancers, including HNSCC. In a recent phase I clinical trial^5^ involving HLA-A2^+^ patients with HNSCC, patients were treated with a multiple CTL and T helper cell-defined, wt sequence p53 peptide-loaded DC-based adjuvant vaccination. The vaccination was shown to have some beneficial effects on the recipients. In patients with advanced HNSCC, however, there were limited post-vaccination anti-wt sequence p53 peptide-specific immunologic responses. Overall, wt sequence p53 peptide-specific CTL frequencies were increased post-vaccination in 69% of patients, with IFN-γ secretion detected in these cells in 25% of patients, but consistently decreased Treg frequencies relative to pre-vaccination values were also observed in these patients. However, disease free survival (DFS) after vaccination did not correlate with the presence or expression levels of p53 in the patients’ tumor cells nor with frequencies of wt sequence p53 peptide-specific CD8^+^ T cells in their peripheral circulation. Despite advances in the developing cancer vaccines, these findings are consistent with the poor clinical responses observed in many previous vaccine-based, cancer immunotherapy studies^9,11^.

To promote further understanding of the nature of wt p53 peptide-specific responses in patients with HNSCC and its relevance to patient survival and p53-based immunotherapy, it is important to determine the frequency and functional activity of wt sequence p53 peptide-specific CTL relative to their differentiation/maturation phenotype in these individuals. T cells have been characterized by their phenotypic and functional profiles into T cell subsets, namely, naïve (TN), central memory (TCM), effector memory (TEM) and terminally differentiated T cells (TTD). One established protocol for identifying these subsets is the differential expression of certain phenotypic markers, such as chemokine receptor 7 (CCR7) and CD45RA^12,13^. In addition, CTL function can also be assessed by monitoring IFNγ production and CD247/perforin expression. TN CD8^+^ T cells (CD45RA^+^CCR7^+^) are activated when interacting with antigen-presenting cells (APC) in secondary lymph nodes and rapidly proliferate and differentiate into TCM (CD45RA^−^CCR7^+^) and TEM (CD45RA^−^CCR7^−^). TEM migrate into the peripheral tissues and efficiently differentiate to effector cells TTD (CD45RA^+^CCR7^−^) while TCM home to the secondary lymphoid organs and retain the ability to proliferate and differentiate into TEM upon T cell receptor stimulation by antigen^12^. In this study, we determined the frequency and phenotype of wt sequence p53 peptide-specific T cells in the peripheral circulation of HLA-A*0201^+^ patients with HNSCC by multicolor flow cytometry using HLA-A*0201 tetramers (tet) complexed with wt sequence p53_264–272_ or p53_149–157_ peptides, referred to as tet_264–272_ and tet_149–157_, respectively. We further evaluated their correlation with clinicopathological factors as well as p53 and HPV status of the patients’ tumor specimens.

## Methods

### Ethics Statement and the criteria of patient inclusion

The clinical sample collection was carried out in accordance with the guidelines and protocols approved by the internal ethics board at University of Pittsburgh Cancer Institute (Pittsburgh, PA), and written informed consent was obtained from each participating individual prior to participation in the study. Patients with HNSCC (n = 33) were selected for inclusion into this study. None were receiving treatment at the time of blood draw. All blood samples were drawn pre-therapeutically after histological confirmation of HNSCC before removal of the cancers. All were HLA-A2^+^, as determined by sero-phenotyping of their peripheral blood mononuclear cells (PBMC) using monoclonal antibodies (mAbs) BB7.2 and MA2.1 (produced by hybridomas obtained from American Type Culture Collection, Manassas, VA)^8^. The clinicopathologic characteristics of the patients are listed in Table 1. TNM classification of malignant tumors according UICC 7th ed was used.

**Table 1:** Clinicopathologic characteristics and frequency of circulating wt p53-specific tet_264–272_^+^ CD8^+^ T cells in 33 patients with HNSCC who donated PBMC for this study.

Characteristics	Patients n (%)	High frequency n (%)	Medium frequency n (%)	Low frequency n (%)	Negative n (%)
Age (years)					
Mean range	42–82				
<60	16 (48)	2 (29)	5 (63)	6 (50)	3 (50)
≥60	17 (52)	5 (71)	3 (37)	6 (50)	3 (50)
Gender					
Male	26 (79)	6 (86)	6 (75)	8 (67)	6 (100)
Female	7 (21)	1 (14)	2 (25)	4 (33)	0 (0)
Primary tumor cite					
Oral cavity	7 (21)	1 (14)	1 (13)	4 (33)	1 (17)
Oropharynx	16 (48)	4 (57)	5 (61)	4 (33)	3 (50)
Larynx	6 (18)	2 (29)	1 (13)	1 (8)	2 (33)
Others	4 (12)	0 (0)	1 (13)	3 (26)	0 (0)
Primary tumor stage^*^					
T1	15 (45)	5 (72)	4 (50)	2 (9)	4 (67)
T2	7 (21)	1 (14)	2 (24)	2 (9)	2 (33)
T3	8 (24)	1 (14)	1 (13)	6 (73)	0 (0)
T4	3 (10)	0 (0)	1 (13)	2 (9)	0 (0)
Nodal status^*^					
N0	16 (48)	6 (86)	5 (63)	3 (26)	2 (33)
N1	7 (21)	0 (0)	2 (25)	2 (9)	3 (50)
N2	10 (30)	1 (14)	1 (12)	7 (65)	1 (17)
Tumor differentiation					
Well	5 (15)	1 (14)	2 (25)	1 (8)	1 (17)
Moderate	24 (73)	6 (86)	6 (75)	9 (74)	3 (49)
Poor	2 (6)	0 (0)	0 (0)	1 (8)	1 (17)
Undifferentiated	2 (6)	0 (0)	0 (0)	1 (8)	1 (17)
Tumor p53 protein accumulation					
Positive	18 (55)	0 (0)	3 (37)	10 (83)	5 (83)
Negative	15 (45)	7 (100)	5 (63)	2 (17)	1 (17)
p16 expression					
Positive	9 (27)	2 (29)	5 (63)	1 (8)	1 (17)
Negative	24 (73)	5 (71)	3 (37)	11 (91)	5 (83)
Therapy					
Surgery only	18 (55)	6 (86)	4 (50)	4 (33)	4 (67)
Surgery + radiation therapy	10 (30)	1 (14)	2 (25)	5 (42)	2 (33)
Surgery + chemoradiotherapy	2 (15)	0 (0)	2 (25)	3 (25)	0 (0)

^*^TNM classification of malignant tumors according UICC 7th ed.

### Collection of peripheral venous blood

Peripheral venous blood (30–50 mL) was drawn into heparinized tubes that were transferred to the laboratory and lymphocyte recovery on Ficoll-Hypaque gradients was immediately conducted. The recovered PBMC were washed, counted, and directly stained for *ex vivo* flow cytometry. The elapsed time between phlebotomy and PBMC staining for flow cytometry was within 2 hours.

### Tetramers, antibodies and staining

The PE-labeled tet_264–272_ and tet_149–157_ reagents were obtained through the National Institute of Allergy and Infectious Diseases Tetramer Facility in Atlanta, GA. Titrations of tetramers and specificity assays were as follows: (a) all tetramers were pre-titered on bulk or cloned CD8^+^ T cell lines with specificity for the wt sequence p53 peptides were available in our laboratories to determine optimal reagent concentrations and to distinguish positive from negative signals; (b) negative controls were used with HLA-A2^+^ PBMC in all assays; (c) a cut-off for tetramer binding to PBMC of HLA-A2^−^ normal donors (n = 10) was established as previously described by us^1^. The lower limit of detection (LLD) was defined as the frequency of 1/7800 cells or approximately 0.01%. This LLD was used as a cut-off for evaluating all tetramer results presented in this manuscript.

The following anti-human mAbs were used in this study: CD8-PC5 (Beckman Coulter, Miami, FL), CD45RA-FITC (Immunotech), CCR7-PE and CD247-APC BD Biosciences perforin-FITC; BD Biosciences, San Jose, CA).

The staining for tetramers and cell surface antigens by flow cytometry was performed as previously described^8^. Briefly, for p53 tet, aliquots of diluted stock (1/100) of tet were added directly to subtly disrupted cell pellets at ambient temperature (5–7 × 10^6^ cells). The cells were incubated for 30 minutes at room temperature in the dark. Next, 5 μl of each mAb was added on the cell subtlety disrupted pellet, followed by 30 minutes incubation at 4 °C in the dark. Cells were washed, centrifuged and resuspended in 500 μl of PBS/0.5% (wt/vol) paraformaldehyde. Flow cytometry was performed within 30 minutes.

### Immunohistochemistry

Immunostaining for p53 protein was performed as previously described^9^. Briefly, formalin-fixed, paraffin-embedded tissue specimens were sectioned (4 μm thick), deparaffinized and rehydrated in a series of graded ethanol. Immunohistochemical staining was performed using a mAb against p53 (DO-7, Dako, Carpinteria, CA, USA), which recognizes an epitope in the N-terminus between amino acid 35 and 45 and reacts with the wt and most mutant forms of p53 protein, followed by the avidin-biotin-peroxidase method to visualize the p53 according to the manufacturer’s instructions (Dako). Positive and negative controls were included in each run for quality control of the immunoreactivity. IgG isotype mAb was used as a negative control. Normal-appearing salivary gland tissue or skeletal muscle from patients with HNSCC served as an internal non-tumor control. A tumor was considered p53 positive when >25% of the tumor cells showed staining intensity of 2+ and higher on a scale of 0–4+. For p16 staining, mouse monoclonal antibody specific for p16 (1:100 dilution, clone DCs-50; neomarkers, Fremont, CA, USA) was used as introduced before^14^. p16 expression was scored as positive if there was strong and diffuse nuclear and cytoplasmic staining in >60% of the tumor. Three independent experienced observers, who were blinded to the patient clinical information, performed semiquantitative evaluation of the slides.

### Statistical analysis

The descriptive statistics were provided using the median/range and box plots. The associations among lymphocyte subsets were tested with the t test or Wilcoxon rank sum test (for two groups) or Kruscal Wallis test (for multiple groups). Reciprocal frequencies of tetramer counts were log transformed (base 10) and tested for differences with the paired t test. Multivariate correlation analysis was performed to determine the relationship between the frequency of p53-specific CTL and clinicopathological parameters. All the reported p-values are based on two-sided tests.

## Results

### Frequencies of tet_264–272_^+^ and tet_149–157_^+^ CD8^+^ T cells in peripheral circulation of HLA-A0201^+^ patients with HNSCC

The frequencies of tet_264–272_^+^ CD8^+^ T cells in the peripheral circulation of 33 HLA-A0201^+^ patients with HNSCC was determined by tetramer-based flow analysis, and in a similar manner, the frequency of tet_149–157_^+^ CD8^+^ T cells in samples obtained from 19 of these 33 patients. Based on the lower limit of detection (LLD) of 1/7800, 27/33 patients had detectable frequencies of tet_264–272_^+^ CD8^+^ T cells in their circulation ranging from 1/7800 to 1/483, with an average frequency of 1/2694. Sufficient sample was obtained from 19/33 patients, all of which had detectable levels of tet_264–272_^+^ CD8^+^ T cells for an additional analysis of tet_149–157_^+^ CD8^+^ T cells. These tet^+^CD8^+^ T cells were detectable at relatively high frequencies ranging from 1/4283 to 1/859 in all 19 samples. In these 19 patients, the average frequency of tet_264–272_^+^ CD8^+^ T cells was 1/4707 (range 1/7798-1/1239) and 1/2130 for tet_149–157_^+^ CD8^+^ T cells (range 1/5492–1/859). The reactivity with these two tetramers correlated (correlation: 0.636, p = 0.003, n = 19).

For further subgroup analysis, the frequencies of tet_264–272_^+^ and tet_149–157_^+^ CD8^+^ T cells detected in the peripheral circulation of patients with HNSCC were divided into 3 groups as follows: high frequency >1/2128; intermediate frequency <1/2128 but >1/4767; low frequency <1/4767. The distribution of high, intermediate and low frequency for each tet^+^CD8^+^ T cell specificity is listed in Table 2. In the group of tet_264–272_^+^CD8^+^ T cells, 6 cases were lower than 1/7800 cut off frequency. All frequencies of tet_149–157_^+^CD8^+^ T cells were higher than 1/7800.

**Table 2:** Distribution of high, intermediate and low frequency for each tetramer^+^CD8^+^ T cell.

	tet_264–272_^+^CD8^+^T cells (n = 33) n (%)	tet_149–157_^+^CD8^+^T cells (n = 19) n (%)
High frequency >1/2128	7 (21.2)	7 (36.8)
Intermediate frequency <1/2128 to > 1/4767	8 (24.2)	11 (57.9)
Low frequency <1/4767 to > 1/7800	12 (36.4)	1 (5.3)
Negative <1/7800	6 (18.2)	0 (0)

### Clinicopathological parameters and frequencies of tet_264–272_^+^ and tet_149–157_^+^ CD8^+^ T cells in peripheral circulation of patients with HNSCC

The distribution of high, intermediate and low frequency for tet_264–272_^+^CD8^+^ T cells regarding to the clinicopathological parameters is listed in Table 1. Frequencies of tet_264–272_^+^CD8^+^ T cells tended to be higher in patients with T1 while those with low frequencies tended to be advanced T4 (Table 1). However, a significant inverse correlation between the frequency of tet_264–272_^+^CD8^+^ T cells and the tumor p53 accumulation (r = −0.637, p < 0.05) was noted with low T cell frequencies in the circulation and a high p53 accumulation at the tumors site, strongly suggesting T cell depletion in patients with p53^+^ tumors. Similarly, a significant inverse correlation (r = −0.813, p < 0.05) was found between the frequency of tet_149–157_^+^CD8^+^ T cells and tumor p53 accumulation. There were no significant correlations between any of other clinical parameters listed in Table 2 and the frequencies of wt sequence p53 peptide-specific CD8^+^ T cells determined in the HNSCC patient’s samples (data not shown).

### Disease-free survival relative to clinicopathology parameters and frequencies of tet_264–272_^+^ and tet_149–157_^+^CD8^+^ T cells in peripheral circulation of patients with HNSCC

**Figure 1:**
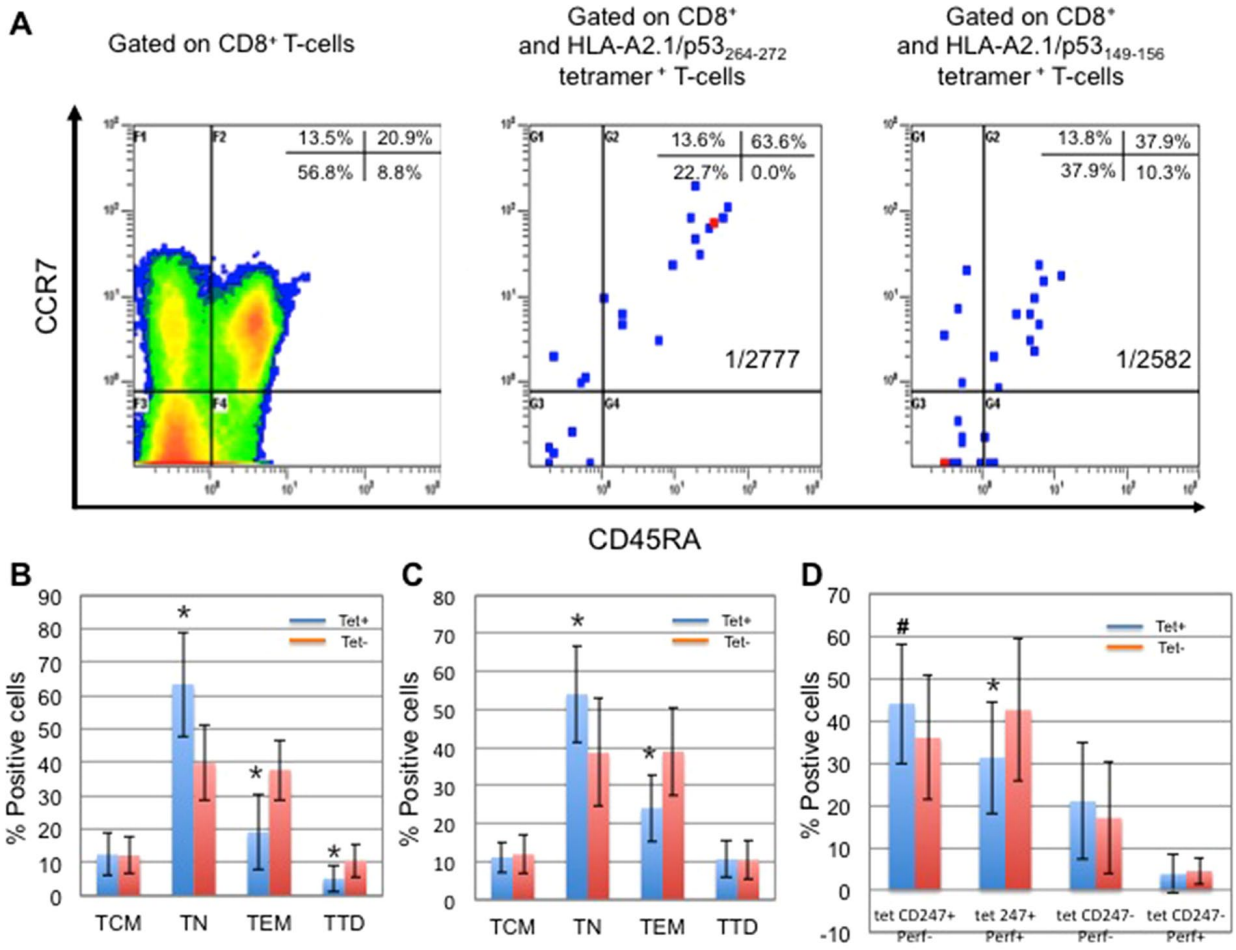
Differential Phenotypes of wt sequence p53 peptide-specific tet^+^CD8^+^ and tet^−^CD8^+^ T cells in the peripheral circulation of patients with head and neck cancer. Examples of dot plots for patient #1 are shown for (**A**) CD45RA and CCR7 expression by wt sequence p53_264–272_-specific or wt sequence p53_149-156_-specific CD8^+^ T cells, and differentiation phenotypes of (**B**) wt sequence p53 tet_264-272_^−^CD8^+^ T cells and wt p53 tet_264–272_^+^CD8^+^ T cells (*p < 0.01), (**C**) wt sequence p53 tet_149–156_^−^CD8^+^ T cells and tet_149–156_^+^CD8^+^ T cells (*p < 0.01), and (**D**) CD247/Perforin expression of wt p53 tet_264–272_^−^CD8^+^ T cells and wt p53 tet_264–272_^+^CD8^+^ T cells (^#^p < 0.05; *p < 0.01).

Among the 33 patients, 14 died of disease (DOD) and 1 died of unknown reason; 17 patients remain alive with no evidence of disease and 1 patient was alive with disease. The median follow-up was 8.82 years (range 2.33–23.42 years). Thus, 3-year DFS was 85% and 5-year DFS was 76% (Fig. 1A**)**. No significant difference in DFS between patients who had p53^+^ versus p53^−^ tumors was observed (Fig. 1B). As expected, HPV p16 status strongly predicted improved clinical outcome even within this small patient group (Fig. 1C**)**. There was no significant difference of DFS, however, among patients who were stratified between those with no or low frequency of tet+ T cells and those with median or high frequency of tetramer reactive T cells (Fig. 1D).

### Differentiation/maturation phenotypes of tet^+^CD8^+^ T cells and tet^−^CD8^+^ T cells in peripheral circulation of patients with HNSCC

**Figure 2:**
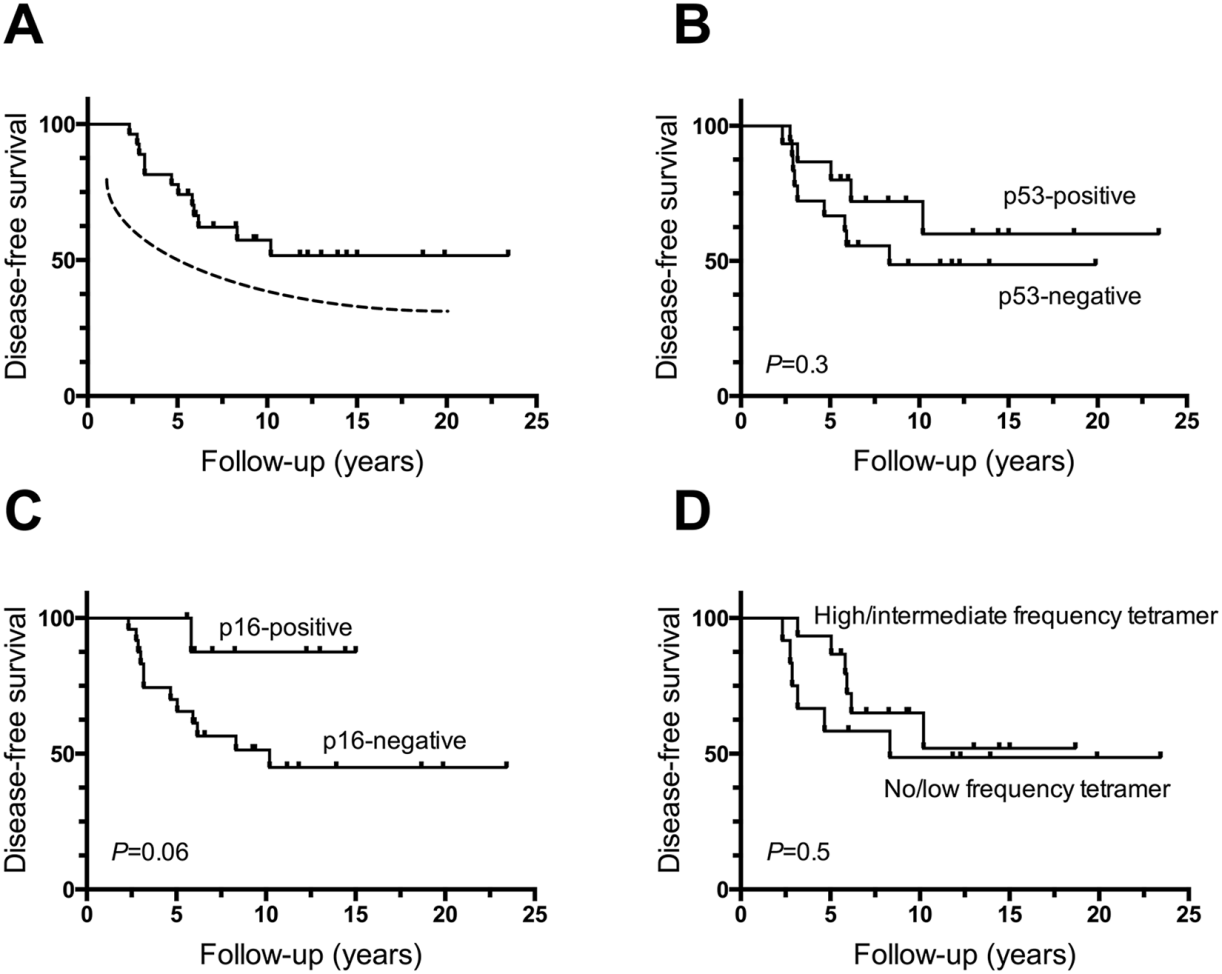
Disease-free survival (DFS) of patients with head and neck cancer. DFS was 85% after 3 years and 76% after 5 years (**A**). DFS for patients based on p53 status (**B**), HPV p16 status (**C**) and the frequency of tetramer wt p53_264–272_^+^CD8^+^ T cells (**D**).

The tet^+^CD8^+^ T cells and tet^−^CD8^+^ T cells were co-stained for T cell surface marker expression CD45RA and CCR7 and analyzed by flow cytometry to determine their differentiation/maturation status as TN, TCM, TEM, and TTD. Representative dot plots obtained from one patient are shown in Fig. 2A.

As shown in Fig. 2B,C, the subpopulation of TN cells contained in both tet^+^ CD8^+^ T cell populations show a significant increase relative to the tet^−^ CD8^+^ populations in the patients’ circulation. For tet_264–272_^+^CD8^+^ it was p < 0.001 and for tet_149–156_^+^CD8^+^ it was p = 0.005. In contrast, TEM tet^+^ CD8^+^ T cells for both specificities are significantly decreased when compared to the tet^−^ population; tet_264–272_^+^CD8^+^ T cells it was p < 0.001 and for tet_149–156_^+^CD8^+^ T cells it was p = 0.001.

TD cell subpopulation was significantly decreased in tet_264–272_^+^CD8^+^ T cell population (p = 0.002) compared to the TD tet^−^ subpopulation. There was no significant difference of TD tet^+^ CD8^+^ T cell subpopulations between tet_149–156_^+^ and tet_t_^−^ CD8^+^ T cell populations in the patients’ samples.

### Comparison of the percentages of the differentiation/maturation phenotypes of the tet_264–272_^+^ and tet_149–157_^+^ CD8^+^ T cell populations and tet^−^ CD8^+^ T cell populations in patients’ peripheral circulation

The median percentages of the four differentiation/maturation phenotypes of tet_264–272_^+^ CD8^+^ T cells were compared to those of tet^−^ CD8^+^ T cells relative to whether the patients’ peripheral circulation had high, medium, low, or no detectable levels of tet_264–272_^+^ CD8^**+**^ T cells in their circulation. The results of this analysis are presented in Table 3. Only in patients with high frequencies of tet_264–272_^+^ CD8^+^ T cells in their peripheral circulation were any significant differences detected: TN cells (CD45RA^+^/CCR7^+^) were significantly increased (p = 0.049), while TD cells (CD45RA^+^/CCR7^−^) were decreased (p = 0.022) (Table 3). No significant findings between phenotypes of tet_149–157_^+^CD8^+^ T cells and tet^−^ CD8^+^ T cell populations were detected (data not shown).

**Table 3:** Association of phenotypes^a^ of wt p53-specific tet_264–272_^+^CD8^+^ or tet_264–272_^−^CD8^+^ T cells with frequencies (n = 27).

	n	TN (CD45RA+/CCR7+)	TEM (CD45RA-/CCR7-)	TCM (CD45RA-/CCR7+)	TD (CD45RA+/CCR7-)
Tet_264–272_^+^CD8^+^					
High frequency	7	76 (46–87)	10 (3–33)	12 (8–17)	2 (0–6)
Intermediate frequency	8	65 (44–91)	22 (3–50)	10 (0–25)	3 (0–7)
Low frequency	12	56 (25–76)	22 (5–35)	14 (0–27)	8 (2–25)
		**P** = **0**.**049**	P = 0.059	P = 0.338	**P** = **0**.**022**
Tet_264–272_^−^CD8^+^					
High frequency	7	38 (19–44)	40 (23–57)	11 (1–21)	10 (6–20)
Intermediate frequency	8	41 (23–55)	38 (22–50)	11 (3–17)	10 (4–22)
Low frequency	12	40 (7–68)	36 (17–65)	13 (3–32)	11 (4–24)
		P = 0.933	P = 0.857	P = 0.917	P = 0.767

^a^The data are median percentages of positive cells among all tet^+^CD8^+^ T cells or all tet^−^CD8^−^ T cells with the ranges in parentheses.

### Association between clinicopathological parameters and differentiation/maturation phenotypes of tet^+^_264–272_ CD8^+^ T cells

Possible associations between clinicopathological parameters and the mean percentage of tet_264–272_^+^ CD8^+^ T cells with different phenotypes detected in the peripheral blood of patients were also investigated (Table 4). A significantly (p = 0.029) lower mean percentage of tet _264–272_^+^ TN CD8^+^ T cells was found in patients with T3–4 stage. In patients without p53 accumulation at the tumor sites, the percentage of tet _264–272_^+^ CD8^+^ TD cells declined (p = 0.03) and a trend for increased TN cells (p = 0.06) was seen. No other significant findings relative to the other clinicopathological parameters studies.

**Table 4:** Associations of clinicopathologic characteristics and phenotypes of circulating wt p53-specific tet_264–272_^+^ CD8^+^ T cells in 27 patients with HNSCC.

Characteristics	n	TN (CD45RA+/CCR7+)	TEM (CD45RA−/CCR7−)	TCM (CD45RA−/CCR7+)	TD (CD45RA+/CCR7−)
**Age (years)**					
<60	13	64	20.6	11.4	4
≥60	14	62.8	17.8	13.4	6.1
		P = 0.9	P = 0.6	P = 0.5	P = 0.39
**Gender**					
Male	20	63.6	19.5	12.4	4.4
Female	7	62.7	17.9	12.1	7.2
		P = 0.9	P = 0.8	P = 0.9	P = 0.3
**Primary tumor site**					
Oral cavity	7	62	19	15	4
Oropharynx	13	68	16	10	6
Larynx	4	69	18	12	1
Others	4	44	31	17	8
		P = 0.12	P = 0.35	P = 0.38	P = 0.34
**Primary tumor stage**					
T1–2	16	66.9	17.3	10.9	4.8
T3–4	11	58.2	21.7	14.5	5.7
		**P** = **0**.**029**	P = 0.45	P = 0.26	P = 0.5
**Nodal status**					
N0	14	67	18	12	3
N1	4	58	24	15	3
N2	9	59	19	12	10
		P = 0.47	P = 0.74	P = 0.76	P = 0.09
**Tumor differentiation**					
Well	4	74	11	12	2.6
Moderate-poor	23	61	20.5	13	5.6
		P = 0.2	P = 0.2	P = 0.9	P = 0.37
**Tumor p53 protein accumulation**					
Positive	13	56.5	22.3	13.5	7.6
Negative	14	69.8	16	11.4	2.8
		P = 0.06	P = 0.25	P = 0.47	**P** = **0**.**03**
**p16 expression**					
Positive	8	69.3	17	9.8	3.8
Negative	19	60.9	19.9	13.5	5.7
		P = 0.28	P = 0.6	P = 0.25	P = 0.47

### Comparison of the differentiation phenotype (CD247/perforin) of tet_274–272_^+^ and tet_149–156_^+^CD8^+^ T cells versus tet^−^ CD8^+^ T cells

Sufficient cells were available from 20/33 patients for additional co-staining of CD8^+^ T cells with tet_274–272_ and CD247/perforin. The percentage of CD247^+^perforin^+^tet_264–272_^+^CD8^+^ T cells was found to significantly decrease while that of CD247^+^perforin^−^tet_264–272_^+^CD8^+^ T cells significantly increased when compared to the CD247^+^perforin^+^ tet^−^ populations (Fig. 1D). Seven patients had increased CD247^−^/CD247^+^ ratio in perforin^+^tet_264–272_^+^CD8^+^ T cells (data not shown). CD247^−^perforin^+^tet_264–272_^+^CD8^+^ T cells were not detected in 11 patients. Two patients had similar percentages of CD247^−^perforin^+^tet_264–272_^+^CD8^+^ T cells as their tet^−^ populations. Moreover, the percentage of CD247^−^perforin^+^tet_264–272_^+^CD8^+^ T cells was correlated to TD tet^+^ cells (p = 0.019). Samples from nine of 20 patients were also available for co-staining with p53_149–156_ tet and CD247/perforin. There were no significant differences in these populations in patients stained with tet_149–156_ (data not shown).

## Discussion

### Wt sequence p53 tet^+^CD8^+^ T cells identified in patients with HNSCC

Consistent to our earlier findings and those of others^9,15^ tet_264–272_^+^ CD8^+^ T cells were identified in the circulation of most, but not all patients participating in this study. Furthermore, in patients who had tet_264–272_^+^ CD8^+^ T cells in their circulation, tet_149–157_^+^ CD8^+^ T cells were also detectable, indicative of a polyclonal reaction of T cells to this tumor antigen. The lack of a wt sequence p53 peptide immune response in some patients observed in this study has been noted in previous ones as well and may, in part, be due to the clonal deletion or anergy of effector T cells specific for self-epitopes^16^ or apoptosis of T-cell receptor (TCR) variable β-chain-restricted antigen-responsive T cells^17^.

### Differentiation/Maturation Phenotypic differences between tet^+^ and tet^−^ CD8^+^ T cells in HNSCC patients’ circulation

In this study, we determined that in patients with a high frequency of tet^+^ CD8^+^ T cells in their peripheral circulation, tet^+^ cells with an N phenotype increased while those with the mature EM and TD phenotypes declined compared to those in the tet^−^ populations. Previously, we have shown that tet^+^ CD8^+^ T cells in HNSCC patients preferentially localized to the tumor sites and tumor-involved lymph nodes and their frequencies increased in the population of tumor-infiltrating lymphocytes (TIL)^8^. Therefore, possible trafficking of tet^+^CD8^+^ T cells with TD and EM phenotype to peripheral sites might alter the phenotype composition of these effectors in the circulation and enhance the N compartment.

CD247 or CD3-ζchain and perforin expression are widely used markers for T cell activation and the expression of perforin was found to decrease in CD247^+^ tet^+^CD8^+^ cells compared to CD247^+^ tet^−^ CD8^+^ T cells. In addition, in some patients, tet^+^CD8^+^ T cells displayed a CD247^−^ phenotype and a strong correlation with a TD phenotype and perforin expression. This observation suggests that CD247^+^ perforin^+^ tet^+^CD8^+^ T cells may have migrated to peripheral sites in these patients concurrent with downregulation of CD247 expression^8^. A better 5-year survival has been shown in patients with tumors infiltrated by TILs expressing normal levels of CD3-ζchain than those showing loss of CD3-ζchain expression^18,19^. Although in the current study, CD247^−^perforin^+^tet^+^CD8^+^ T cells were not correlated to the low frequency tet^+^CD8^+^ T cells, it suggests that TD cells that traffic to the peripheral site lack T cell receptor-ζchain and may account for the unsuccessful or limited immune response in some patients.

### Frequencies of circulating tet^+^CD8^+^ T cells negatively correlated to p53 expression in tumor tissues

In this study, patients who had high frequency of tet^+^ CD8^+^ T cells were found to have lower p53 accumulations in their tumor tissues while patients with low frequency of tet^+^CD8^+^ T cells had higher p53 accumulations in tumor tissues. This discrepancy is consistent with our previous finding relating frequencies of these tet^+^ CD8^+^ T cells in the peripheral circulation of HNSCC patients and p53 mutational sites in their tumors^9^ and suggests that these CTL in responsive patients could have eliminated tumor cells capable of processing and presenting the targeted epitope resulting in the immunoselection and outgrowth of “epitope-loss” tumors.

### Clinical relevance of circulating tet^+^ CD8^+^ T cells

The frequency of circulating tet^+^ CD8^+^ T cells was found to decrease in HNSCC patients with advanced disease. Furthermore, a decline of the N phenotype subpopulation but similar TD phenotype subpopulation in tet^+^CD8^+^ T cells was found in advanced T stage as compared to lower T stage. The reason for this correlation remains presently unknown. As previously shown, the presence and frequency of tet^+^ CD8^+^ T cells among TIL did not correlate with tumor stage indicating it was independent of tumor progression in HNSCC^8^. We speculate that patients with advanced T stage HNSCC may not present the wt sequence p53 peptide epitopes properly or have limited CTL recognition due to the downregulation of expression of antigen-processing machinery components^17,20^. For example, CTL function may be suppressed by Treg, which are abundant in TIL at tumor sites^8,21^. Treg frequency, which is responsible for immunosuppression of adaptive and innate immunity and correlates with tumor progression and outcome, increases in patients with HNSCC^8,22^.

An important parameter that contributes to the complexity of analyzing immune responses of patients to HNSCC is HPV infection, since High Risk-HPV-related HNSCC currently accounts for 25% of HNSCC and up to 70% of oropharyngeal squamous carcinoma^23^. HPV E6 binds and degrades the p53 while E7 binds and degrades the pRB retinoblastoma tumor suppressor protein^24^. In HPV^+^ HNSCC, circulating HPV E7 specific CD8^+^ T cells are detectable indicating that endogenously-induced E7-specific immunity exists in these patients^7^. It has also been shown that wt and mutant p53 molecules are sensitive to HPV E6-mediated degradation and *in vitro* and *in vivo* results in increased presentation of the wt sequence p53_264–272_ peptide by HLA-A•0201^+^ tumors for CTL recognition^6^. Using p16 as the marker of HPV infection, there was no correlation of HPV expression in patients’ tumors to the frequencies of tet^+^ CD8^+^ T cells, in the patients’ circulation. However, consistent with current literature, p16 expression did correlate with improved DFS.

Relevant to the results of this study are the findings obtained from the recent multiepitope wt sequence p53 peptide vaccine clinical trial^5^, the vaccination of patients with HNSCC resulted in an increased frequency of tet_264–272_^+^ CD8^+^ T cells in their peripheral circulation and decreased levels of Treg. Additionally, circulating tet^+^ CD8^+^ T cells were more vulnerable to spontaneous apoptosis suggesting their preferential demise represents a mechanism of immunoescape of tumor cells^2^. Nonetheless, these patients had a favorable two-year DFS of 88% as compared to 70% of DFS in a similar clinical trial cohort including unvaccinated patients treated with chemoradiation^25^. Interestingly but unaccounted for at present, was that a limited and weak post-vaccination, wt sequence p53 peptide-specific immunity was observed in 5/16 patients. Overall, the presence or expression levels of p53 in these patients’ tumor tissues and the presence of tet^+^ CD8^+^ T cells in their peripheral blood after vaccination did not correlate to DFS. The explanation for this phenomenon is still unclear and requires a more extensive analysis of the nature of the immune response to wt sequence p53 peptide- specific epitopes in patients with HNSCC.

In perspective, our data support findings from the interaction of other solid cancers with the immune system: presence of a range of tumor-specific T cells in various differentiation stages and an immunosuppressive tumor microenvironment on multiple levels^8,26^. With the advent of checkpoint inhibition treatment, it appears that tumor-specific T cells can be activated more readily and tumor immune suppression ameliorated to some extent. It will be intriguing to see if the empirically observed clinical benefit of checkpoint inhibitors^27,28^ can be explained by the use of our T cell characterization methods and that they could be used as a read out of patient response or selection for treatment.

In summary, the results of this study further revealed the complex nature of wt sequence p53 peptide-specific immune responses in HNSCC patients and highlight several new parameters, like naïve T cell activation by vaccination and checkpoint inhibition by modulating the PD-1/PD-L1 axis, that should be considered in developing the design and analysis of future vaccination protocols to enhance the efficacy of p53-based immunotherapy of HNSCC.
